# Perceptions and patient care needs among hepatitis B patients during COVID-19

**DOI:** 10.1186/s12913-022-08153-5

**Published:** 2022-06-30

**Authors:** Sherrie Flynt Wallington, Min Jeong Jeon, T. Angeline Nguyen, Choosonjargal Byambaa, Y. Tony Yang, Daisy Le

**Affiliations:** 1 School of Nursing, George Washington University, Washington, DC USA; 2 Hepatitis B Initiative of Washington DC, Washington, DC USA

**Keywords:** HBV, Hepatitis B virus, COVID-19, Patients, Prevention, Resources, Racial and ethnic minorities

## Abstract

**Background:**

The novel coronavirus of 2019 (COVID-19) has been and continues to be a rapidly developing public health crisis, that has also disrupted routine and maintenance health care for people living with chronic conditions. Some of these chronic conditions also put individuals at increased risk of COVID-19 complications, particularly if the condition is not under control. For these reasons, the exploratory study reported here examined the needs and preparedness of patients at a community health organization that specifically provides hepatitis B virus (HBV) care for high-risk groups that had previously tested positive for HBV.

**Methods:**

Current study utilized exploratory analysis of qualitative COVID-19-related statements collected during calls to a total of 44 patients reached during April and May, 2020 in the Washington D.C. area. Researchers worked with a community based non-profit organization to reach current HBV + and HCV + patients to provide retention in care and assess patient needs in maintaining management of their condition adapted to include offering medication refills, telehealth, and other resources. We gathered emergent themes, using socio-ecological framework, regarding capacity and needs for managing their chronic condition in a vulnerable population during the initial, most interrupted, time period of a global public health crisis.

**Results:**

From the notes of the calls, five thematic categories emerged: COVID-19 prevention awareness, assistance program access, medical resource access, access to knowledge and awareness about assistance programs, and needs and barriers. From these five themes, providers can develop strategies to better prepare their patients and provide care to patients with chronic conditions during major disruptions.

**Conclusions:**

Future recommendations include increasing hepatitis and COVID-19 vaccine efforts, collaborating with community partners, and screening and understanding social determinants of health that affect racial and ethnic minorities.

## Background

The novel coronavirus of 2019 (COVID-19) has been and continues to be a rapidly developing global public health crisis. What initially presented as primarily a respiratory virus with a high mortality rate, has more recently come to be understood to be a systemic disease that causes inflammation and cellular death in many parts of the body, leading in some cases to long-term impact and suffering [1–3]. Several underlying conditions contribute to an increased risk for long-term COVID-related complications, including advanced age (even for healthy elders), obesity (even for healthy individuals), cancer, chronic kidney disease, chronic lung diseases, neurological conditions (e.g., dementia, Alzheimer’s), diabetes, Down syndrome, heart conditions, HIV infection, otherwise immunocompromised, chronic liver diseases (including hepatitis B virus (HBV)), pregnancy/recently pregnant, sickle cell disease and thalassemia, smoking or having smoked, organ or blood transfer recipient, having survived a stroke or cerebrovascular disease, and substance use disorders. We also know that having an underlying condition that is not well controlled puts one at a higher risk than if it is well-managed [4, 5], and that some of these conditions tend to come in clusters (a syndemic) including HBV, often presenting with HIV and substance use disorders [6]. People with one or more of these conditions are at significantly increased risk of hospitalization, intubation, and mortality if they contract COVID-19.

Many of these underlying conditions are disproportionately distributed among marginalized and minority communities, putting already economically vulnerable populations at greater risk [7, 8]. Globally, COVID-19 has increased disparities, both directly and through disruptions to health care and other necessary services [9]. Across the United States (U.S.), among the COVID-19 reporting that includes race/ethnicity, we see that people of color are significantly more likely to die of COVID-19 than white patients [10, 11]. Black or African American patients have died at a rate of 174 per 100,000 (as of March 7, 2021), compared to 124 per 100,000 white patients. In the District of Columbia, while Black or African American individuals represent 46% of the population and 49% of the cases, they represented 76% of the deaths, compared to white individuals who comprise 41% of the district’s population, and 10% of the deaths [11].

In the case of HBV as an underlying condition, we also see disparities. Globally, particularly in low- and middle-income countries, where we see significantly higher burden of disease in parts of Africa and Asia, COVID-19 has caused disruptions to already vulnerable efforts to detect, prevent, and treat HBV [12]. Domestically in the U.S., until very recently, non-Hispanic Asian and Black populations were among the most impacted by HBV [13]. A significant effort has been made to vaccinate (particularly infants and small children), and screen pregnant people (to avoid parent/infant transmission), which has greatly decreased the HBV rates across the country over the last 20 years. However, screening for the virus among adults is suboptimal [14]. While non-Hispanic Black and non-Hispanic white folks are reported to have HBV in similar rates (1.0 per 100,000 population), mortality from it is not equal. Non-Hispanic Black men die from it at a rate of 2.7 per 100,000 with the disease, compared to 1.6 per 100,000 of non-Hispanic white men. Non-Hispanic Black women die at a rate of 1.0 per 100,000, compared to non-Hispanic white women 0.9 per 100,000 [15]. (Does not report on mortality for Asians or other races.) As higher mortality suggests the disease is not under control, this suggests that there is work to be done in supporting better management of the disease generally, and that this is urgently needed in the context of COVID-19’s increased risks.

To compound this increased urgency in need, when COVID-19 first overtook the U.S. in March 2020, most non-urgent medical care (as well as other activities) were temporarily suspended [16, 17]. As of June, 2020, an estimated 41% of U.S. adults had delayed or avoided medical care, both routine and emergency [18]. As management of chronic conditions requires regular and consistent medical care, there is concern that this interruption, as crucial as it was, may have negatively impacted at-risk populations. As new variants of COVID-19 emerge, and infectious disease experts continue to warn that another pandemic is probable [19–22], there is a risk of repeated interruptions to care. Thus, it is important that HBV patients be prepared for all situations, including limited access to necessities. To make sure patients are sufficiently prepared, it is important to understand their starting point. This study utilized a regular patient outreach communication channel during the early months of COVID-19 in the United States (March through May of 2020) to explore preparedness for managing their HBV condition within the COVID-19 pandemic context, and their understanding of and ability to manage their COVID-19 risk level.

## Methods

### Approach

This study is an exploratory analysis of qualitative COVID-19-related statements collected during calls to hepatitis B patients. Researchers worked with Hepatitis B Initiative of Washington DC (HBI-DC), a community based non-profit organization that “works to prevent liver cancer caused by hepatitis B and C among people of Asian, Pacific Islander, and African descent, and other high-risk groups” [23]. During March through May of 2020, their regular outreach to current HBV + and HCV + (hepatitis B and C virus positive) patients to provide retention in care and assess patient needs in maintaining management of their condition adapted to include offering medication refills, telehealth, and other resources. The research presented here uses qualitative data analysis to gather emergent themes regarding capacity and needs for managing their chronic condition in a vulnerable population during the initial, most interrupted, time period of a global public health crisis.

Patients were asked the following questions related to COVID-19:

COVID-19 Risks and Prevention:

- Are you at risk for COVID-19? Yes/No
- Do you have any symptoms? Yes/No
  - ◦ If Yes: Have you recently travelled to an area with a known local spread of COVID-19?
  - ◦ If Yes: Have you come into close contact (within 6 feet) of someone who has a laboratory confirmed COVID-19 diagnosis in the past 14 days?
  - ◦ If Yes: Do you have fever (greater than 100.4F or 38.0C) OR symptoms of lower respiratory illness, such as coughing, shortness of breath, difficulty breathing, or sore throat?
  - ◦ If Yes: Have you been able to get tested? Yes/No
    - ▪ If Yes: What was the result?
- Do you need any support or resources? Yes/No
  - ◦ If Yes: What kind?

Financial Hardship & Social determinants of Health:

- Are you or family member (spouse, partner) experiencing a financial hardship (i.e. loss of job, reduction of work hours) due to the COVID-19? Yes/No
- Are you able to pay your mortgage or rent or utilities? Yes/No
- Do you have enough food? Yes/No

### Theoretical framework

The Socio-Ecological Model illustrates how an individual’s health choices are influenced by layers of factors at intrapersonal, interpersonal, social network, institutional, and community levels [24]. In particular this study looks at how institutional and community factors impact individuals’ follow-through on care management. At the community level, what services and resources were missing or harder to access during the shut-downs? At the institutional level, what did patients want and/or need from the clinic they were receiving care from?

### Setting

HBI-DC called patients who had tested positive for hepatitis B or hepatitis C at their screening events. This DC-based non-profit organization works with individuals of Asian, Pacific Islander, and African ancestry and other high-risk groups to provide hepatitis B and hepatitis C education, screening, vaccination, and linkage to care. Patients were reached by phone between March 23^rd^ through May 30^th^, 2020, by HBI-DC staff.

### Participants

Participants were current patients at HBI-DC, that had a current hepatitis B or C positive test result, and that had provided a phone number. All patients were adults and of Asian, Pacific Island, or African descent. Forty-four patients were reached during those two months, which is comparable to the outreach efforts from previous years. Of those reached, 27 were male (61.4%), and the average age was 51.3 years old (range 31–74 years old). Of those reached, 31 (70.5%) reported not having insurance, and 35 (79.5%) reported not having a primary care provider (PCP) (Table 1.)

**Table 1:** Participants

Patients who were reached:	44
Male:	27 (61.4%)
Female:	17 (38.6%)
Average age:	51.3 years
Age range:	31 to 74 years
HBV Ags Positive:	44 (100%)
Reported not having insurance:	31 (70.5%)
Reported not having a PCP:	35 (79.5%)

### Data collection

Staff at HBI-DC used the phone numbers that patients provided to contact patients with a positive HBV test result. The phone calls were conducted, and notes were taken by the staff.

### Data analysis

De-identified notes were individually coded using NVivo by two members of the study team, and data were entered into NVivo to prepare for coding. Based on the theoretical framework and study goals, an initial code book was developed, with new codes being added as needed. The initial coding process involved reading the notes, one by one, and marking keywords in context on each note. During open coding, the constant comparative approach was used to group the codes into categories and identify themes [25–27].

Axial coding was then applied to look at the inter-relationship of themes. Repetitive words and phrases were placed together under selected categories, or *nodes*. Nodes allow the researcher to gather related material in one place to look for emerging patterns and ideas. Each node was based on the codes developed in analysis, which were guided by the theoretical model and study goals. Intercoder reliability was assessed using Kappa statistics, and was 1.0. The Kappa coefficient is a measure of agreement between raters or measurement procedures for categorical data, and a value of 1.0 indicates perfect agreement [28].

## Results

Based on the call notes, five thematic categories emerged (Table 2):

**Table 2:** Emergent Themes and Supporting Patient Statements

Emergent Theme: Example Quotes
**Awareness of COVID-19 Protective Measures:**
“During this quarantine time she stays home as directed and only leaves for groceries and knows the precautions to help decrease exposure (wearing masks and washing hands).”
“Reminded him to wash hands regularly and wear a mask.”
**Access to Assistance Programs Offered:**
“He told me of how his taxi business is experiencing hardship since COVID. It is only one cab, he is a sole owner. [Patient] also explained that he did not know where to go for help. I researched both small business and unemployment benefits. I called the small business administration and they told me they are only issuing loans. I felt unemployment benefits was best for him as he did not want a loan. I texted him at 2:00 the numbers for both DC AND XXX county unemployment.”
“Can't file unemployment or stimulus check. Advised to watch Tuguldur TV on Facebook or Youtube every day for 10–15 min about updates in Mongolian language.”
“Per Doctor, patient had Obamacare and now due to new job, lost insurance. Doctor suggested reapplying due to COVID and he might qualify, resource was sent to patient.”
“[Caller] will research on Aunt Bertha. Can offer resource on free bus tokens or she can apply for Medicaid and medical transportation is free.”
“He says he needs assistance with paying his electric bill, so I will find him a resource to help him. Referred him to Friends for Neighborhood Progress Inc to get assistance with electric bill. I spoke with a representative with the organization and they will help him.”
“Needs assistance with receiving unemployment benefits, got confused with website so am calling unemployment to get her help with filling out the form. She also needs a free clinic to go to for Hepatitis.”
**Access to Medical Resources:**
“Patient … checks up with Doctor every year, needs medication but too expensive, advised her to call Doctor and ask for generic that insurance will cover because she said it's $1,000 a month. She also has an order for an ultrasound but waiting after this pandemic to go.”
“Missed last appointment. Can't remember if in January. Not taking medications. Advised TeleHealth is available.”
“She also told me she has an MRI of the brain scheduled for May but cancelled it because she wasn't comfortable going into Kaiser during this time.”
“Doctor also said his friend was being treated by Korean doctor through HBI-DC (although we don't know who that is) so he needed his results to get treatment.”
“5/8/20 Will need a Swahili interpreter to make the appt as she really does not understand. … 5/12/20: Doctor's office does have language line. He will try to find Swahili speaker in office. We suggested that someone from office call patient to make an appointment because worried patient won’t be able to navigate prompts on system.”
“He would like to get help to find a doctor to follow up. And his wife is interested in information about future screen events.”
**Access to Knowledge and Information on Assistance Programs:**
“… I researched both small business and unemployment benefits. I called the small business administration and they told me they are only issuing loans. I felt unemployment benefits was best for him as he did not want a loan. I texted him at 2:00 the numbers for both DC AND XXX county unemployment.”
“Can't file unemployment or stimulus check. Advised to watch Tuguldur TV on Facebook or Youtube every day for 10–15 min about updates in Mongolian language.”
“[Caller] will research on Aunt Bertha. Can offer resource on free bus tokens or she can apply for Medicaid and medical transportation is free.”
“He says he needs assistance with paying his electric bill, so I will find him a resource to help him. Referred him to Friends for Neighborhood Progress Inc to get assistance with electric bill. I spoke with a representative with the organization and they will help him.”
“Needs assistance with receiving unemployment benefits, got confused with website so am calling unemployment to get her help with filling out the form. She also needs a free clinic to go to for Hepatitis.”
**Needs and Barriers:**
“Patient … needs medication but too expensive, advised her to call Doctor and ask for generic that insurance will cover because she said it's $1,000 a month. She also has an order for an ultrasound but waiting after this pandemic to go.”
“[Spoke with patient’s daughter]. Patient went to Mongolia, taking meds, medications are finishing. Called Doctor for meds, doctor said NIH pharmacy can ship to Mongolia. She has doctor's cell phone. Other medications for keeping normal pulse are finishing too. The HBV meds are finishing mid-May. Advised to call doctor … They tried calling, no success. Found doctor's email address and texted to patient's daughter.”
“…he says he could not remember taking a test with us.”
“Spoke with the patient and he states that he never heard anything about Hep B from the clinic only Hep C.”
“… patient had Obamacare and now due to new job, lost insurance.”
“5/8/20 Will need a Swahili interpreter to make the appt as she really does not understand. … 5/12/20: Doctor's office does have language line. He will try to find Swahili speaker in office. We suggested that someone from office call patient to make an appointment because worried patient won’t be able to navigate prompts on system.”
“She has a hard time getting to the clinic as it is far, and would like to see if we can send her to a clinic closer. … Will let patient know that since she is getting free treatment and care, going twice a year might be the best option for her, especially because they have been following her and her history. [Coordinator] will ask if she has Medicaid/Medicare because there are more options for free transportation. In the meantime, will research on Aunt Bertha. Can offer resource on free bus tokens or she can apply for Medicaid and medical transportation is free.”
“… patient still has not been to doctors, does not seemed too concerned since he's had it since childhood, he also says he's too lazy to go and has many friends and family members who have Hep B and they're fine, some on meds and some aren't, he seems concerned about side effects of medications…”
“He just got a Ultrasound test from his family doctor on 5/15/20 because of the pain in the stomach. But he didn't got the result yet. He would like get some information about how to get free or reduce cost medication. He doesn't have insurance and have financial hardship to get treatment.”
“Needs help with food assistance and would like us to find him a Doctor near Frederick as Baltimore is too far. Sent him the information through Aunt Bertha for food stamps.”

### Awareness of COVID-19 protective measures

**Fig. 1:**
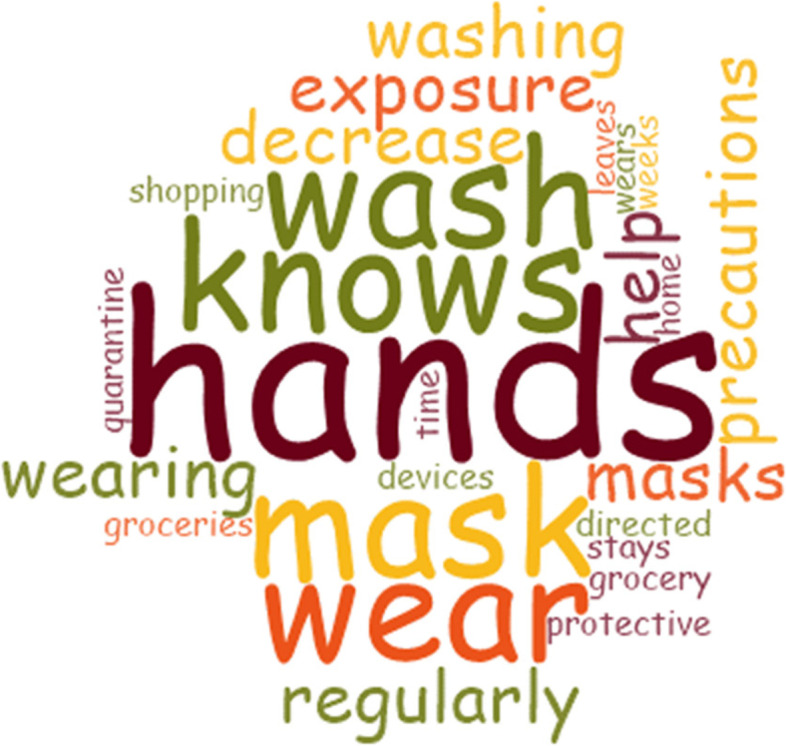
COVID-19 Prevention Word Cloud. This word cloud shows the prevalence of the words used by participants to explain measures they have put in place to help in preventing COVID-19 infection

There was a level of awareness when it came to protective measures to prevent COVID-19 infection. They knew the preventative steps they needed to take, such as wearing a mask, social distancing and frequent handwashing. In the calls, some reminders were also made to the patients to ensure they stayed safe and prevented the spread of COVID-19, “Reminded him to wash hands regularly and wear a mask…” (Fig. 1).

### Access to assistance programs offered

There were various programs in place to support people financially, medically, with food and transportation. People could access their stimulus check through the mail to help boost them financially. There were participants who had lost their jobs due to the COVID-19 pandemic and were required to file for unemployment in order to receive financial support.

In terms of food assistance, there were new, COVID-specific places where people could receive free food for specific periods of time. Those who had no information about these food assistance programs were informed about them through these medical follow-up calls. For instance, there were schools that offered meal programs to replace free school lunches for children that depend on that resource. These resources helped families who had less resources to survive on, “Told that schools are offering free meals, his kids are college students…” There was also a provision of food stamps program through Aunt Bertha Assistance and Resources website (now known as findhelp.org) and this information was shared with patients as well, “…sent him the information through Aunt Bertha for food stamps.”

For medical assistance, medication was being mailed to the patients wherever they were to ensure continuity in medication uptake, “Called her to inquire about meds and she said they have the meds mailed to patients. I called patient back and gave her number to patient to call her right away.” This service was being made available even to patients who have moved regardless of their new location, “…patient went to Mongolia, taking meds, medications are finishing. Called Doctor for meds, doctor said NIH (National Institutes of Health) pharmacy can ship to Mongolia.” There was also the provision of telehealth services for patients in need of doctor’s appointment that could be accessed by phone, “Offered that TeleHealth is an option to have a video call with her doctor…” Information was also given to patients to help them update insurance coverage, “Doctor suggested reapplying due to COVID and he might qualify; resource was sent to patient.”

The patients were also offered free transportation services for medical purposes for those patients who had no means of transportation to get medical attention, “Can offer resource on free bus tokens or she can apply for Medicaid and medical transportation is free.”

### Access to medical resources

There were various medical resources that were availed to the patients. Access to these resources varied from patient to patient. There were those who were able to access health screening services such as colonoscopy, ultrasound, endoscopy and blood tests, “He had a PCP. He just got an Ultrasound test from his family doctor on 5/15/20 because of the pain in the stomach.” Unfortunately, there were those who were not able to access these services due to the restricted movement during the pandemic, “Patient was scheduled for a liver biopsy on 3/18, cancelled due to COVID-19 situation…” There were patients who also needed access to free medical care, “She also needs a free clinic to go to for Hepatitis…” and they were advised on how to go about it.

Access to the health facility was not a challenge for some of the patients, while others were not able to access the health facility. One of the reasons mentioned for lack of access was the long distance between the patient and the health facility, “Patient says Baltimore is too far.” For some, the facility wasn’t open for appointments, “I assisted him with tips as he has pre-diabetes and he is waiting to get to his Doctor as it is closed due to COVID.”

Patients were able to see their doctors, and their appointments were scheduled at different times throughout the year. There were patients with appointments every three or six months and others who had appointments scheduled as frequent as weekly. However, for most of the regular check-ups, appointments were cancelled due to the COVID-19 pandemic, “Patient went to NIH every week for testing to get ready for treatment to start as soon as approved, since January. April 10 appointment was cancelled due to COVID situation…” There were patients who had been appointed different doctors from their regular ones, which was a barrier for some, “…contacted patient coordinator and got Dr's e-mail address. Hasn't sent e-mail, a little hesitant, because used to have a different doctor…”.

When it comes to medication uptake, most of the patients were reported to be adhering to the prescriptions given to them by their doctors, “Spoke with patient and he was put on medication from December 2019 and has finished his prescription.” Few were reported to not adhering to the prescriptions and not taking medication at all, “Not taking medications.” Another statement made about this was, “Patient says she is still taking meds…”.

### Access to knowledge and information about assistance programs

Information about the available resources and programs in place to assist patients in various ways was known to some and unknown to others. Information for local support services for food assistance, financial assistance, etc., was provided to those patients that did not already have the information. One of the statements made about food assistance was, “Patient started receiving food stamps. Searched for free school lunch in her area, advised to go on Monday to elementary school for free lunch for her child….”

The patients were also given information concerning where they will be able to get help with paying utility bills for those not able to, “He says he needs assistance with paying his electric bill, so I will find him a resource to help him. Referred him to Friends for Neighborhood Progress Inc to get assistance with electric bill.”

Some patients also requested information about how to file for unemployment in order to get financial assistance, “He also stated that he would like for me to send him information for unemployment assistance in Prince William County and SNAP (Supplemental Nutrition Assistance Program)…” The most convenient support information was shared with the patients after thorough consideration and research:

*“He explained that he did not know where to go for help. I researched both small business and unemployment benefits. I called the small business administration and they told me they are only issuing loans. I felt unemployment benefits was best for him as he did not want a loan.”*

When it came to healthcare information, some of the patients were given information on how they could get follow-up appointments because they were not aware of how to do that and asked for assistance, “He would like to get some information about doctor follow up and medication treatment…”.

Information was also given to the patients about how they could manage their existing health conditions because they were not able to access a doctor due to COVID-19, “I assisted him with tips as he has prediabetes and he is waiting to get to his doctor as it is closed due to COVID.” Importance of taking medication and going for regular medical check-ups were also emphasized with the patients.

### Needs and barriers

There were various challenges and barriers that the patients were facing which were identified during the follow-up calls. Unemployment was one major impact of COVID-19 which affected patients leading to financial barriers. Some were not able to pay for their utility bills or even buy food for their families, “…needs assistance with rent (connected) because working reduced hours, 4–5 h/day.” There were requests for information concerning food assistance from the patients, “Needs help with food assistance…”.

There were also challenges with medication access. There were patients who had no money to purchase medication, “…needs medication but too expensive, advised her to call doctor and ask for generic that insurance will cover because she said it's $1,000 a month.” For some, their medication was running out and needed a refill but there were a few challenges regarding picking up the medication:

*“I asked her if she has enough medications and she says she will need a refill soon in the next two weeks, since she goes to Kaiser, I advised her to call soon to ask for a refill because they might have procedures for pick up due to the Coronavirus.”*

Some of the patients also mentioned not having received information about their illnesses, “…spoke with the patient and he states that he never heard anything about Hep B from the clinic only Hep C…” and arrangements were made to assist them. Transportation also came up as a need mentioned by one of the patients, “…patient needs help with transportation…”. They were assisted with information regarding this, too.

**Fig. 2:**
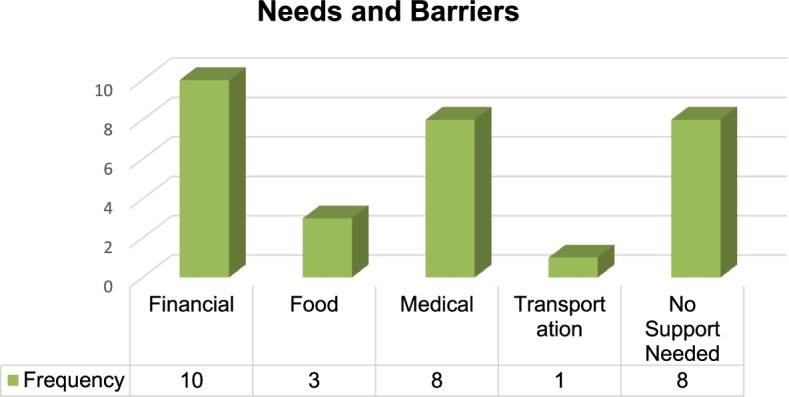
Primary Needs and Barriers Reported by Participants. This bar graph shows the frequency of some of the barriers faced by the participants during the COVID-19 pandemic

**Fig. 3:**
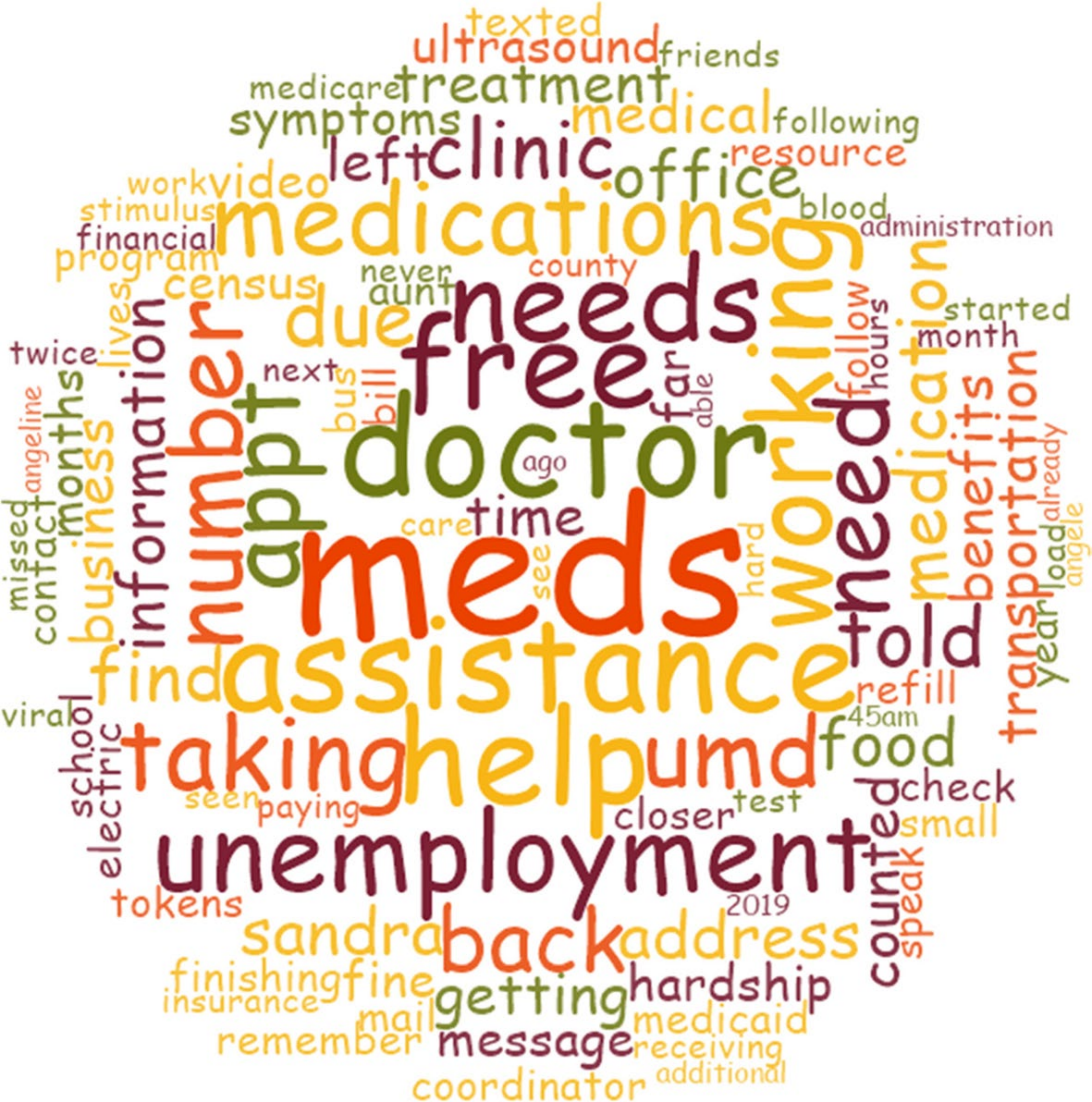
Needs and Barriers Word Cloud. This word cloud shows the words used to express some of the barriers and challenges the participants were facing

In addition, there were patients who did not have any challenges or barriers stopping them from accessing the services and products they required for healthy living, which was noted in quite a number of the statement reports, “Patient seems to be doing good and has no need for assistance…” (Figs. 2 and 3).

## Discussion

The study reported here explored the needs of patients affected with Hepatitis B virus during the early months of the COVID-19 pandemic. The following five themes emerged from exploratory analysis of the qualitative interviews from 44 participants: 1) awareness of COVID-19 protective measures, 2) access to assistance programs offered, 3) access to medical resources, 4) access to knowledge and information about assistance programs, and 5) needs and barriers.

Participants were aware and reminded of precautions to stay safe from COVID-19 due to their increased risk for complications with positive Hepatitis B virus infection status. During routine outreach from HBI-DC staff, participants were informed of assistance programs, such as school food programs; medical support including ensuring continuation of medication as prescribed and providing guidance for telehealth with a provider as needed; and transportation services for medical care. In terms of medical resources available for participants, while some were able to receive screenings and blood tests as necessary, some procedures were cancelled due to prioritization of care for patients directly affected by COVID-19. Similarly, participants described varied access to healthcare facility and appointments that were reassigned to a different provider or cancelled due to COVID-19. Information about assistance programs for food, utilities, unemployment, financial support, and transportation were provided to participants who were not aware of the resources available for them. Assistance with medical follow-up appointments and emphasis on adherence to prescribed medication regimen was provided for those having a hard time getting access to care due to COVID-19. Some participants described barriers to medication access due to financial hardship and not receiving necessary information or medical advice about their hepatitis diagnosis due to COVID-19.

Most participants expressed a strong need for assistance, and challenging barriers to accessing care and basic necessities (e.g., food, transportation), as well as insufficient knowledge about medical and assistance programs. It is important to highlight that all 44 participants were of Asian, Pacific Island, or African descent. Kardashian, et al., (2021) emphasize social determinants of health and heightened liver disease disparities during COVID-19 [29]. Racial and ethnic minorities as well as non-U.S. born foreign nationals are most often affected by chronic liver disease and related adverse outcomes [29]. Upstream determinants of health, such as social distancing measures, as well as personal financial hardship due to unemployment following COVID-19, made it difficult for many to access health care. Kardashian et al. (2021) highlight the impact of COVID-19 on liver disease disparities especially for those experiencing low-income, food insecurity, delays in treatment and screening, limited availability of community support groups, loss of a job and/or health insurance, homelessness, as well as systematic racism and bias [29]. Due to an increased risk for adverse outcomes from COVID-19, and vulnerability to COVID-19 infection for those already affected by hepatitis infection, people were compromising other health-related choices, including being less willing to go grocery shopping which increased food insecurity, and delaying health care [29]. It is also important to highlight that 70.5% of the participants reported not having insurance and 79.5% reported not having a PCP. Kardashian et al., (2021) report that racial and ethnic minorities who were already experiencing poverty prior to COVID-19 had experienced a lack of support (e.g., social network, community assistant programs), medication shortage, and access to health care due to unemployment and financial hardship [29]. Our study explored many of the same questions that theirs did and found many of the same emergent themes in data collection from communities with multiple layers of vulnerability.

Based on the needs highlighted from our study, as well as the literature, future recommendations include:

- Increasing COVID-19 vaccine efforts especially for racial and ethnic minorities to decrease the disparities of impact from the disease.
- Collaborating with community partners to address needs of vulnerable populations affected by hepatitis infection, including providing information about how to use telemedicine, and assistance programs for medication, transportation, food, etc.
- Screening for, understanding, and addressing social determinants of health that affect racial and ethnic minorities.

The strengths of the study reported here are that we were able to collect qualitative exploratory data from 44 individuals affected by hepatitis infection via a community partner outreach communication channel during the early part of 2020, when the COVID-19 began to emerge in the U.S. This allowed us to get an understanding of how COVID-19 was impacting the participants and their basic care needs during the that stage of the pandemic.

Some of the limitations of the study are that it is a relatively small study, consisting of 44 participants utilizing a single community health organization in the Washington D.C. area. There were only participants of Asian, Pacific Island, or African descent, excluding Hispanics who are also largely affected by liver diseases [29]. Future studies should consider exploring experiences of other racial and ethnic minority groups including Hispanics, as well as gathering information from community health centers across the U.S., and longitudinal studies to capture the needs and barriers of patients affected by hepatitis infection during a pandemic that now has affected people worldwide for almost 2 years.

## Conclusion

**Fig. 4:**
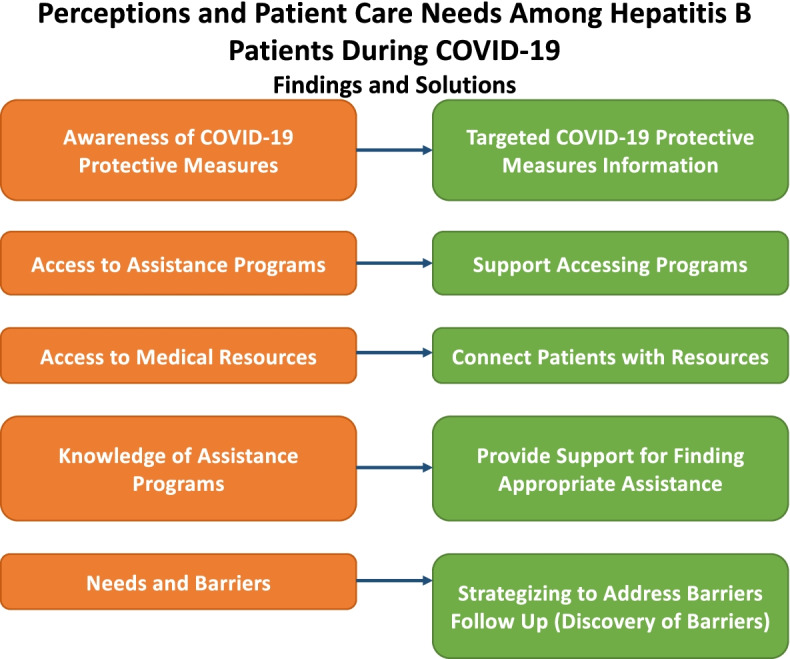
Needs-Based Themes and Potential Solutions. This figure illustrates how the themes that emerged from our data analysis translate to potential practices that better support patients through a disruptive public health crisis

This current exploratory qualitative study examined the needs and preparedness of patients at a community health organization that specifically provides HBV care for high-risk groups that had previously tested positive for HBV. From the notes of the calls, health care providers can develop strategies to better prepare their patients and provide care to their patients during major disruptions. Needs-related themes that emerged in this study indicate potential solutions (see Fig. 4.) For example, patients being unclear on COVID-19 protective measures suggest that the relevant public health messages were not reaching their communities at that time. Patients needed access to assistance programs and medical resources, even knowledge of existing assistance programs related to their specific needs. Providing that information, and supporting patients accessing those resources are indicated. Within the theme of needs and barriers, each patient had different challenges, suggesting the need for one-on-one strategizing to help patients overcome those barriers. The role of follow up in discovering these barriers and needs was also highlighted.

Due to the novelty of the COVID-19 global pandemic, in early 2020, health care providers found themselves needing to pivot their care delivery models and priorities without precedence and limited guidance. At the time of publication two years later, there is still very limited published guidance on how best to provide support for patients with chronic conditions during a public health crisis. In looking at the impact of COVID-19 on diabetes patients, another chronic condition associated with increased risks and similar maintenance needs, suggested recommendations are similar to the ones found in this study. These include clear guidance communication, continuity of care, and access to services and resources [30]. Our recommendations are in alignment with the US Department of Health and Human Services *Viral Hepatitis National Strategic Plan*’s recommendations in light of COVID-19, to address the added barriers to care that are faced by communities that shoulder a disproportionate share of the disease burden by addressing informational and resource access deficiencies [6].

## Data Availability

The datasets generated and/or analyzed during the current study are not publicly available due to the privacy of research participants but are available from the corresponding author on reasonable request.

## Abbreviations

HBI-DC: Hepatitis B Initiative of Washington DC
COVID or COVID-19: Coronavirus of 2019
U.S.: United States
Hep B or HBV: Hepatitis B Virus
HBV+: Hepatitis B Virus positive
HBV Ags: Hepatitis B Virus Surface Antigens
Hep C or HCV: Hepatitis C Virus
HCV+: Hepatitis C Virus positive
NIH: National Institutes of Health
PCP: Primary care provider
SNAP: Supplemental Nutrition Assistance Program
